# Tooth enamel oxygen “isoscapes” show a high degree of human mobility in prehistoric Britain

**DOI:** 10.1038/srep34986

**Published:** 2016-10-07

**Authors:** Maura Pellegrini, John Pouncett, Mandy Jay, Mike Parker Pearson, Michael P. Richards

**Affiliations:** 1 Research Laboratory for Archaeology and the History of Art, University of Oxford, Oxford, OX1 3QY, UK; 2 Department of Human Evolution, Max Planck Institute for Evolutionary Anthropology, Leipzig, 04103, Germany; 3 Division of Archaeological, Geographical and Environmental Sciences, University of Bradford, Bradford, BD7 1DP, UK; 4 Department of Archaeology, University of Durham, Durham, DH1 3LE, UK; 5 Department of Archaeology, University of Sheffield, Sheffield, S1 4ET, UK; 6 UCL Institute of Archaeology, University College London, London, WC1H 0PY, UK; 7 Department of Archaeology, Simon Fraser University, Burnaby, B.C., V5A 1S6, Canada

## Abstract

A geostatistical model to predict human skeletal oxygen isotope values (*δ*^18^O_p_) in Britain is presented here based on a new dataset of Chalcolithic and Early Bronze Age human teeth. The spatial statistics which underpin this model allow the identification of individuals interpreted as ‘non-local’ to the areas where they were buried (spatial outliers). A marked variation in *δ*^18^O_p_ is observed in several areas, including the Stonehenge region, the Peak District, and the Yorkshire Wolds, suggesting a high degree of human mobility. These areas, rich in funerary and ceremonial monuments, may have formed focal points for people, some of whom would have travelled long distances, ultimately being buried there. The dataset and model represent a baseline for future archaeological studies, avoiding the complex conversions from skeletal to water *δ*^18^O values–a process known to be problematic.

Understanding and reconstructing the mobility of past populations and individuals is important in archaeological and forensic studies. One of the ways to address this topic is through the chemical analysis of skeletal remains, for the life history of an individual is recorded in the chemistry and isotopes of his or her body tissues. The use of isotope geochemistry techniques to trace mobility of individuals^1^,^2^,^3^,^4^,^5^,^6^,^7^ relies on the fact that the chemical composition of human (and animal) tissues is acquired principally through ingested food and drink, and the isotopic composition of these items is in turn determined by local climate and environmental conditions. Isotope ratios such as ^18^O/^16^O and ^87^Sr/^86^Sr are employed in soft and hard tissues to investigate origins and mobility of past populations. The relationship between the isotopic composition of local environments and the different biological tissues varies with the type of element and isotopes investigated, the tissue, and often the type of animal species^8^,^9^. The partitioning of isotopes in the environment is, in turn, contingent on many factors, including the geological nature of the substrate, environmental conditions, soil type, hydrological circulation, amount of precipitation, plant species and their distribution^2^,^8^,^9^.

Oxygen isotopes in human hard tissues are commonly used in archaeological and forensic science to study residential changes between childhood and adulthood^10^,^11^,^12^,^13^. Here, and for the rest of the paper, oxygen isotope ratios (^18^O/^16^O) are expressed with the delta (*δ*) notation as *δ*^18^O per mil (‰), where *δ* = *R*_*sa*_*/R*_*st*_–1, *R* being the isotopic ratio, *sa* the sample and *st* the reference standard. In obligate drinkers, such as is the case for humans, oxygen isotope values (*δ*^18^O) in bones and teeth are related to those of local water (rain- and groundwater). Longinelli and Peretti Padalino^14^ demonstrated that a direct relationship exists between the *δ*^18^O of drinking water and the *δ*^18^O of blood water in mice and humans. Following these observations, Longinelli^15^ and Luz *et al*.^16^ found that a linear correlation also exists between the *δ*^18^O of human skeletal bone phosphate (*δ*^18^O_p_) and the mean annual precipitation (*δ*^18^O_w_) characteristic of the area where the individual lived. This correlation is explained by the fact that the mean annual isotopic value of the precipitation falling on a certain area is similar to that of plant food and water available to individuals dwelling in the area. If the nutrients are locally sourced, their isotopic composition is reflected in that of the body water. Since biological apatite precipitates in near equilibrium with body water, it follows that the bioapatite isotopic composition is ultimately correlated with that of local environmental waters. Equally, soft tissue *δ*^18^O values are correlated with those of local waters.

Ehleringher *et al*.^2^ demonstrate that mobility of modern individuals can be traced by means of the *δ*^18^O and *δ*^2^H of hair keratin. These authors provide a useful theoretical and visual method to predict *δ*^2^H and *δ*^18^O values of human hair by mapping the expected values in a Geographical Information System (GIS) platform. Their predictive model is based on expected tap water values from across different areas of the USA and the mass balance between the dietary oxygen and hydrogen isotope inputs from a general “supermarket diet”, i.e. a modern globalised diet. In a comparable approach, we have used the isotopic characteristics of skeletal remains to identify human mobility of past human populations by mapping the variation in tooth enamel *δ*^18^O_p_ of archaeological remains coming from across Britain. The resultant map is based on the expectation that prehistoric people would have sourced their dietary and drink supplies locally, and that these sources are compositionally related to the mean isotopic values of rain and groundwater. This “*isoscapes*” (isotopes in the landscape) approach is novel in archaeology.

## Environmental-based Approaches to Mobility

Many isotopic studies that have employed *δ*^18^O_p_ to trace human mobility in archaeological or forensic contexts have focused on measuring the values of single individuals to compare them with the isotope ranges of local waters expected at the site of recovery^12^,^17^,^18^,^19^,^20^,^21^. When such isotopic values differ from the ones expected, individuals have been considered ‘non-locals’ to the area^13^. Some studies have further attempted to source individuals to specific places of origin, or places where they would have spent their childhood (i.e. the period during their tooth mineralization). However, one of the main difficulties with this process is discerning between the many areas with the same combination of isotopes across the landscape, both within any one country, and across different countries^22^.

Standard practice for investigating mobility of past people with isotopes is to measure the isotopic composition in the body tissues (for example ^18^O/^16^O) and to compare this composition with the distribution of the same isotopes in the local and wider environment. In most archaeological studies the material chosen for analysis is the skeleton, which is the tissue most prone to survive in the burial environment. Bioapatite phosphate and carbonate oxygen isotopes (*δ*^18^O_p_ or *δ*^18^O_c_), measured in teeth or bones, are used to trace human mobility, by converting these values to their equivalent water *δ*^18^O_w_ values according to specific conversion equations available in the literature^15^,^16^,^23^,^24^,^25^. These equations take into account the physiological effects during the integration of the different isotopes into the tissues. The “converted” values so calculated are subsequently compared to rainwater or groundwater patterns of variation, either obtained from the literature^26^,^27^,^28^,^29^ or extrapolated from the general distributions of isotope ratios in precipitations^30^. With this method, non-local individuals are identified based on the difference of their skeletal *δ*^18^O values (converted to water values) from that of the drinking water in their place of recovery. It must be considered, however, that the isotope composition of the same elements in the environment and in living individuals does not vary in exactly the same way. The relationship between the measured value in an individual and the value expected on the basis of the place of recovery is therefore not straightforward, and the biological values recorded in local populations may be more or less consistent with those predicted. Apart from the possibility that someone in the group is not local to the area, the variability in oxygen isotopes recorded in people living in a specific area could be due to several other factors:

1. Some individuals may have been affected by short-term climate conditions (warmer/colder, wetter/drier periods) occurring during childhood formation of their teeth. This may lead to atypical *δ*^18^O values (^18^O-enriched or ^18^O-depleted). Mean annual water values, with which these are compared, are averaged over a long period of time (normally 10 to 30 years), a period which is longer than that required for the tooth to mineralise;
2. Sourcing drinking water from reservoirs other than the local groundwater, for example from rivers coming from higher latitudes or from lakes or ponds, may also contribute to altering the individuals’ expected skeletal *δ*^18^O values compared to the local water values, causing depletion or enrichment respectively in ^18^O;
3. Preparation/treatment of food and water can also contribute to offset skeletal *δ*^18^O values from those expected. Boiling, brewing and cooking practices all cause shifts in the values typical of fresh food and drink from a certain area. These manipulations often tend to produce enrichment in ^18^O^23^,^31^;
4. Finally, analytical problems or errors associated with the mathematical conversion from *δ*^18^O_p_ to *δ*^18^O_w_ may lead to additional modifications of the expected water values^24^.

These factors can all contribute to altering the direct relationship between individuals’ oxygen isotope ratios and the environmental ratios of their place of origin. The best approach is therefore to avoid the conversion of skeletal *δ*^18^O_p_ to water *δ*^18^O_w_, and instead compare the skeletal values directly with other human phosphate values.

This paper provides a distribution model for human tooth enamel *δ*^18^O_p_ values across Britain and describes its most likely variation during the Chalcolithic-Early Bronze Age. The map of variation produced is a predictive model of skeletal *δ*^18^O_p_ values across the region and will allow a direct evaluation of *δ*^18^O_p_ measured in individuals of comparable age, with no need to convert their *δ*^18^O_p_ values to water *δ*^18^O_w_ values. The model assumes that the majority of the samples investigated represent individuals that were “local” to the place of recovery. It also assumes that sampled humans did not engage much in dietary behaviours such as drinking stewed or brewed beverages, which would modify substantially their *δ*^18^O_p_ values or, if they did, they would do similarly at the population level. Finally, it is assumed that environmental conditions remained consistent over the period of the Chalcolithic and Early Bronze Age.

## A Geostatistical Approach to Mobility

**Figure 1:**
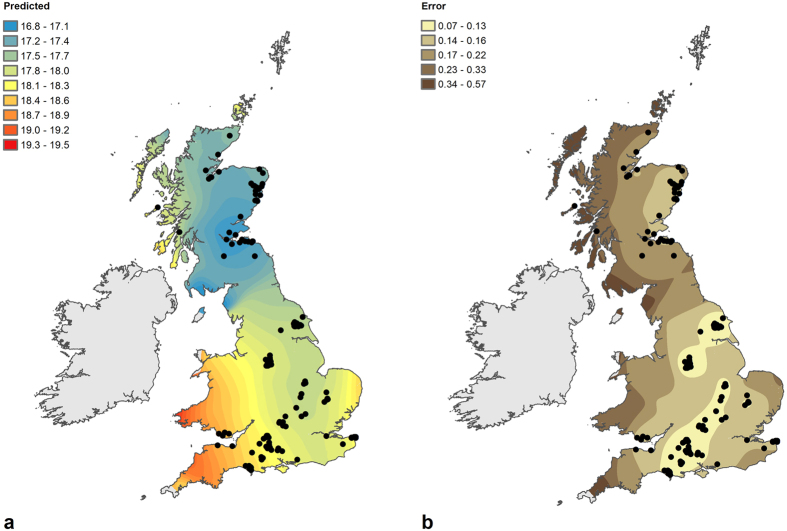
Prediction surface (**a**) and standard error surface (**b**) of the spatial variation in *δ*^18^O_p_ values calculated using Ordinary Kriging (Prediction errors: n = 258; mean = 0.001; RMS = 0.563; standardised mean = 0.001; standardised RMS = 1.002; average standard error = 0.516). All values were retained for coincident points or multiple samples from the same site. Symbology for prediction surface and standard error surface classified using defined intervals and geometric intervals respectively. Ordinary Kriging carried out using the Geostatistical Analyst extension for ArcGIS for Desktop 10.3.1 (http://desktop.arcgis.com/en/arcmap/10.3/guide-books/extensions/geostatistical-analyst/understanding-ordinary-kriging.htm) © EuroGeographics for the administrative boundaries.

The identification of human mobility based on isotope compositions is contingent upon characterisation of the spatial variation of the expected isotope values. Without adequate characterisation of this spatial dimension, it is not possible to identify individuals who are ‘local’ to an area and those who are not with any degree of certainty. Teeth from 261 individuals (denoted by the prefix SK) were sampled in order to characterise spatial variation in human skeletal *δ*^18^O_p_ for the Chalcolithic and Early Bronze Age (2500–1500 BC) as part of the Beaker People Project^7^. The individuals analysed include 22 samples that fall outside Chalcolithic-Early Bronze Age period (see Supplementary Table S1). All are from Britain except for SK 315 (Burial 30, Mound of Hostages, Tara). This, the only sample from the Republic of Ireland, has been described in detail elsewhere^32^ and is excluded from the modelling below. The sample locations indicated in Fig. 1 reflect a number of geographic biases that must be acknowledged prior to undertaking any form of spatial analysis:

1. Sample locations correspond to the places where individuals were buried rather than the places where they lived: in some cases, Chalcolithic and Early Bronze Age settlements may have been located several kilometres from the place of burial^33^;
2. Many of the skeletal samples were recovered from burial mounds excavated by nineteenth-century antiquaries, the locations of which were sometimes poorly recorded. Sample locations were recorded as 4-figure National Grid References, i.e. they were located to the nearest 1km grid square–the highest level of precision common to all samples;
3. Most samples were recovered from areas with geology favourable to the preservation of human bones (e.g. chalk, limestone). Skeletal remains tend not to survive in acidic soils such as those in much of Wales and Scotland. The majority of the Scottish samples included in this data set survived due to the burial rite, which involved a stone cist and avoided soil contact^7^.
4. The resultant dataset exhibits preferential sampling due to the survival of skeletal remains, the availability of material from museum collections, and the areas in which excavators and antiquaries were active. A large number of samples are from the south of England and the northeast of Scotland, with very few samples from the north and centre of Wales and the south and west of Scotland.

Given the complexity of the relationship between *δ*^18^O_p_ and *δ*^18^O_w_, the identification of individuals who are ‘local’ or ‘non-local’ to a particular area is dependent here upon the comparison of the *δ*^18^O_p_ values with all the samples from that area. Preliminary examination of the *δ*^18^O_p_ values reveals marked variability within geographic regions, most notably among the samples from the Highlands and Islands, Yorkshire and the Humber, the East Midlands and the South West–the regions from which the highest number of samples were recovered. The magnitude of the variability within regions is illustrated by samples taken from multiple individuals at Woodhenge (South West–SK 308 and 309), Bee Low (East Midlands–SK200, 210, 216, 221 and 222) and Garton Slack 37 (Yorkshire and the Humber–SK106, 107, 123 and 124), where the range of *δ*^18^O_p_ values for each of these locations was more than half of that for the whole of Britain. Despite this variability–a defining characteristic of the spatial variation in *δ*^18^O_p_ values that should not be ignored or played down–the data exhibit positive spatial autocorrelation at the 99% confidence level. The Moran’s I test statistic, a global measure of spatial autocorrelation, was calculated for the *δ*^18^O_p_ values for the individuals sampled as part of the Beaker People Project (I = 0.315) and compared to the expected value (I_E_ = −0.004). The z-score (z = 6.004) and p-value (p = 0.000) indicate that the test statistic is significant at the 99% confidence level. Spatial autocorrelation, the assumption that the values of nearby samples are closely related to one another^34^, is a pre-requisite of the geostatistical techniques that underpin the approach used in this paper.

Cluster/outlier analysis was carried out in order to characterise spatial patterning in the *δ*^18^O_p_ values. Anselin’s Local Moran’s I test statistics were calculated for each of the samples, with positive values of the test statistic indicating similarity and negative values indicating dissimilarity^35^. Spatial relationships between neighbouring features were defined by inverse distance, with a threshold of c.85km (the distance at which each sample had at least one neighbour). Row standardization was applied to the spatial weights used in the calculation of the test statistic for each sample. Corrections based on the False Discovery Rate were applied to the classification of samples corresponding to clusters/outliers^36^. The values of the test statistic are used to identify samples that form part of a cluster of high/low values (HH/LL) and samples that correspond to outliers with unusually high/low values (HL/LH). The cluster/outlier analysis (see Supplementary Fig. S1) reveals an underlying trend in the *δ*^18^O_p_ values, with clusters of high values in the south and west of Britain (Wales and South West) and clusters of low values in the northeast of Britain (Eastern Scotland and North Eastern Scotland). Two outliers, samples with unusually high or low *δ*^18^O_p_ values, were also identified; namely the samples from Woodhenge (South West) SK308 and 309. These samples, which could be interpreted as samples from ‘non-local’ individuals, were excluded from further analysis.

A continuous surface predicting the spatial variation in *δ*^18^O_p_ values (Fig. 1a) was subsequently generated using Ordinary Kriging–a geostatistical interpolation technique that estimates values for non-sampled locations based on weighted averages of the values of nearby samples^37^,^38^,^39^. Sample locations were de-clustered in order to compensate for any biases introduced by preferential sampling. The underlying trend identified through cluster/outlier analysis was modelled using a 2^nd^ order polynomial trend surface. This trend was subtracted from the data, with the semi-variance modelled on the residuals, and added back in at the end of the interpolation process. The semi-variance was modelled using a stable model (nugget = 0.299684, partial sill = 0.015628, range = 121,384.818021 and parameter = 1.170313), with a smoothed search neighbourhood (smoothing factor = 0.5). Cross-validation of the model, with samples excluded on a case-by-case basis and the observed values compared to the value predicted from the remaining samples, indicates that the model is accurate and unbiased. The standard error surface derived from cross-validation (Fig. 1b) indicates that the predicted values are typically a good fit to the measured values in locations from which multiple samples were taken.

Cluster/Outlier analysis and Ordinary Kriging were carried out using ArcGIS 10.3.1. The parameters used to model the trend and semi-variance were optimised using the Geostatistical Wizard which forms part of the Geostatistical Analyst extension. Geostatistical Analyst supports Ordinary Kriging using either a constant mean or a deterministic trend.

## Spatial Variation in *δ* ^18^O_p_ values

The *δ*^18^O_p_ values measured in the samples range between 16.2‰ and 19.5‰, with a mean value of 17.8‰ (±0.7). The Shapiro-Wilk test statistic (W = 0.992; p = 0.144) indicate that the oxygen values are normally distributed at the 95% confidence level. For 63 individuals from Chalcolithic and Early Bronze Age graves, Evans *et al*.^40^ report a range of 16.2‰ to 19.1‰, with a mean of 17.7‰ (±0.7). The two datasets are statistically indistinguishable. The minor difference at the higher end of the ranges could be due to the larger sample size in this study or differences in the spatial distributions of samples from the respective studies.

**Figure 2:**
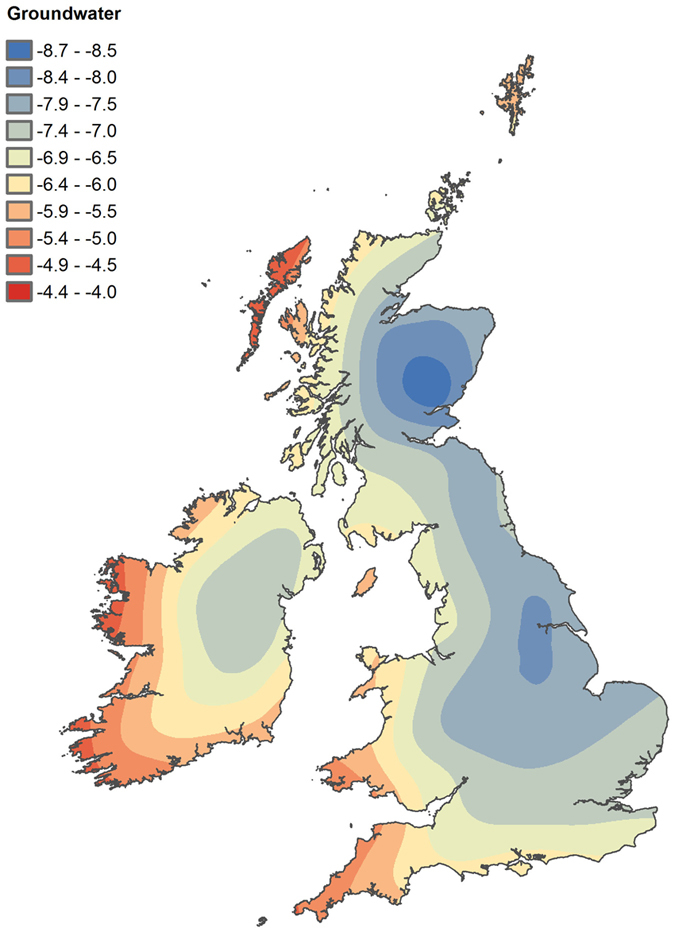
Groundwater oxygen isotope ratio distribution values from Britain and Ireland, showing the decrease in *δ*^18^O values from southwest to northeast (modified from Darling *et al*.^26^).

The underlying trend in the point data, with high values in the south and west of Britain and low values in the northeast of Britain, is clearly evident within the prediction surface (Fig. 1). This trend is broadly in line with that recorded for the rain and groundwater oxygen isotope ratio distribution for Britain and Ireland^26^,^41^. In Britain, rain and groundwater *δ*^18^O values decrease from south to north and from west to east, due to the continuous depletions of air masses as they move over the region. A digitised version of the groundwater isotope map is provided in Fig. 2. The gradient of depletion in ^18^O is quite steep in the western regions, where there is also the highest amount of rainfall, and more gentle towards the eastern regions. Provided that the climate conditions and air mass circulations have not changed substantially in the Holocene, as suggested by Darling *et al*.^26^, this map should provide a good representation of the natural waters available to individuals who inhabited these areas. As stated, individuals who lived in one place for their entire life and who obtained water and food from local sources should have tissue isotope ratios similar to that of the local environmental water (plus or minus a physiological fractionation factor, which is accounted for in the conversion equations^15^,^16^,^23^,^24^,^25^). Theoretically, if all of the individuals sampled for the Beaker People Project had been local, and unaffected by any of the factors described above, the geographic trend in the distribution of skeletal *δ*^18^O_p_ values should mirror that of the water (*δ*^18^O_w_). Although the general trend of depletion in *δ*^18^O values observed for the natural waters (Fig. 2) broadly persists in the skeletal isotopes (Fig. 1a), key differences can be seen between the modelled spatial variation of *δ*^18^O_w_ and *δ*^18^O_p_:

1. The distribution of *δ*^18^O values for phosphate shows a marked difference between northern and southern regions of Britain–a difference that is not observed in the distribution of *δ*^18^O values for water. Particularly, the south–north gradient of variation of *δ*^18^O is more extreme for phosphate than for water–the *δ*^18^O_p_ values in central and southern regions being more ^18^O-enriched than expected;
2. The east–west gradient of variation of *δ*^18^O is more varied for phosphate than water–the *δ*^18^O_p_ gradient for central and southern Britain is in fact attenuated compared to that of *δ*^18^O_w_, while the gradient in the *δ*^18^O_p_ for northern Britain is similar to that of the *δ*^18^O_w_;

These key differences can in part be attributed to factors that may affect the relationship between isotope ratios in living populations and the environment, as well as biases in the spatial distribution of samples discussed above. However, the overall variation in the spatial distribution of *δ*^18^O_p_ suggests that something else besides the physiological/environmental offsets between the local water and the skeletal isotopic values is playing a role.

Already, the two individuals with unusually high (*δ*^18^O enriched) or low (*δ*^18^O depleted) values identified through cluster/outlier analysis (SK308 and 309) and excluded from the modelling of the spatial variation in *δ*^18^O_p_ values can be interpreted as ‘non-local’ to the Stonehenge area in which they were buried. The individuals that form part of the isolated clusters of low values at Staxton (Yorkshire and Humber–SK271) and St. Stephen’s College (South East–SK230, 233 and 234) could also potentially be interpreted as ‘non-local’ to the areas where they were buried (Fig. S1). Three further samples (SK270, 272 and 273) from Staxton also have low *δ*^18^O_p_ values, and the Anselin’s Local Moran’s I test statistics indicate that their *δ*^18^O_p_ values are similar to that of SK271.

Although it is tempting to infer that these individuals were immigrants who brought Beaker pottery to Britain from continental Europe, any notion of a mass migration of the Beaker people is overly simplistic. In fact, the Woodhenge burials date to long after the ‘first-wave’ arrival of Beakers; SK 272 from Staxton also dates to later than the start of the Beaker package^7^ and other burials did not contain this form of pottery. Instead of representing ‘first-wave’ migrants from continental Europe, the outliers and isolated clusters can be considered to reflect a high degree of mobility during the Chalcolithic and Early Bronze Age–with movement both within Britain and between Britain and the Continent. This helps to place in context the apparent long-distance mobility of the so-called Amesbury Archer, a Beaker individual previously identified on the basis of his *δ*^18^O_p_ value as a continental migrant to Britain, although not quite early enough to have arrived in a first wave^42^. The results of the Beaker People Project indicate that his long-distance mobility was exceptional rather than typical of these patterns of movement^6^.

**Figure 3:**
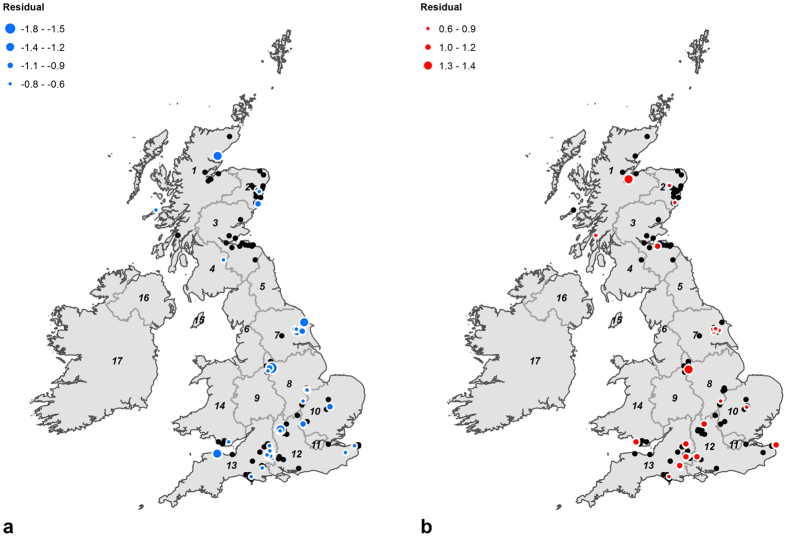
Plot of the positive (a-^18^O-enriched values) and negative (b-^18^O-depleted values) residuals from cross-validation of the geostatistical model, showing the inter-regional variability in *δ*^18^O_p_ values incremented in steps of 0.3‰–the most reported measurement error in oxygen isotope analysis. Positive and negative residuals are plotted separately to minimise the impact of overlapping symbols and highlight that samples with both ^18^O-enriched and ^18^O-depleted values occur in the same regions (Region codes: 1 Highlands and Islands, 2 North Eastern Scotland, 3 Eastern Scotland, 4 South Western Scotland, 5 North East, 6 North West, 7 Yorkshire and the Humber, 8 East Midlands, 9 West Midlands, 10 East of England, 11 London, 12 South East, 13 South West, 14 Wales, 15 Isle of Man, 16 Northern Ireland, 17 Republic of Ireland.). Cross-validation carried out using the Geostatistical Analyst extension for ArcGIS for Desktop 10.3.1 (http://desktop.arcgis.com/en/arcmap/10.3/guide-books/extensions/geostatistical-analyst/performing-cross-validation-and-validation.htm) © EuroGeographics for the administrative boundaries.

The underlying trend in predicted values and small standard prediction errors (Fig. 1) masks the variability identified in the measured values of samples from the same region. Plotting the residuals from cross-validation of the geostatistical model, i.e. the differences between the predicted and measured values for each sample (Fig. 3), highlights the magnitude of this inter-regional variability. Samples with large residuals (^18^O-depleted <−0.6‰ and >^18^O-enriched 0.6‰, equivalent to over 2σ of the measurement error) are found in many areas, including the Stonehenge region (South West), the Peak District (East Midlands and West Midlands) and the Yorkshire Wolds (Yorkshire and the Humber). The large residuals for the samples from the Stonehenge region, the Peak District and the Yorkshire Wolds are comparable to those in samples recovered from Medieval ports and cities, based on forthcoming geostatistical modelling of data published by Evans *et al*.^40^. Each of the highlighted areas is rich in Chalcolithic and Early Bronze Age funerary and ceremonial monuments that would have formed focal points or gathering places for people, some travelling long distances and ending up being buried there.

The spatial outliers can therefore be regarded as non-local to the regions that they were found and, by extension, the high degree of variability identified in the Yorkshire Wolds, the Peak District and the Stonehenge region can perhaps best be accounted for by a high degree of population mobility in those areas.

## Conclusion

This paper presents a geostatistical model of spatial variation in the distribution of human phosphate oxygen isotope values (*δ*^18^O_p_) for Chalcolithic and Early Bronze Age Britain. The model can be employed to study the characteristics of local human populations and the mobility of individuals, and provides a baseline against which future samples from archaeological burials can be compared, without the need to undertake complex conversions of the data from skeletal to water *δ*^18^O values, a process known to be problematic. This model helped to identify *δ*^18^O_p_ outliers, i.e. individuals that are statistically too different from the rest of the population to be ‘local’, and can be used to determine whether measured values from new samples are ‘local’ or ‘non-local’ to the area from which they were recovered. The hypothesis underpinning this model is that the majority of people living in a certain area are not immigrants. It should be noted that the dataset used to create our geostatistical model is not complete and does not include data from all geographic regions. However, the dataset can be supplemented with samples from other regions, where available, once the offsets resulting from the lack of internationally-recognised materials for phosphate oxygen isotope analysis and commonly-agreed protocols for precipitation or calibration techniques are better understood. Although specifically created for Britain, the model can be exported to other settings or isotopes. The approach adopted in this study can be applied to datasets from other geographical regions or chronological periods. Based on oxygen isotopes alone (or the isotopes of any other individual element), it is not possible to distinguish, definitely, immigrants from local people if they have grown up in environments with similar isotope compositions to the place of recovery. It is therefore advisable to combine oxygen isotope profiles with other isotopic profiles, and/or other archaeological information, to be able to spot immigrants that can resemble locals based on one of their isotopic ratios. Other isotope measurements (Sr and S) are available for the individuals included in the Beaker People Project and are published separately^6^,^7^,^43^.

## Methods and Materials

The model of *δ*^18^O_p_ variation was created from a new database of individual teeth coming from Chalcolithic and Early Bronze Age human burials in Britain and Ireland. Most of this collection dates from 2500 to 1500 BC^6^,^7^,^44^ and many are individuals that had been buried with Beakers, a type of pottery that appeared in Britain and Ireland shortly after 2500 BC^45^. A total number of 261 individual teeth were analysed. Tooth enamel fragments of about 20 mg were sampled from each tooth at the Division of Archaeology of the University of Bradford, prepared and precipitated as silver phosphate (Ag_3_PO_4_) at the Department of Human Evolution-Max Planck Institute for Evolutionary Anthropology in Leipzig (DE), and measured for their *δ*^18^O_p_ value back at the University of Bradford. Second permanent molar teeth were preferentially chosen although, in a few cases, when this tooth was not available, premolar or other teeth were used (SK51, SK53, SK129, SK135). Permanent molar teeth (except first molars) and premolars mineralize over a comparable period of time, approximately from 2–3 years of age to about 8, thus sensibly longer than one year^46^,^47^. The teeth chosen target a very early stage of the individual’s life, i.e. childhood. The time span covered by the tooth mineralization should provide a good indication of the mean annual environmental conditions of the area where the individual grew up and take into account inter-annual climate variability. First molars were avoided for their likelihood of being characterized by breastfeeding isotopic signatures^48^. The sampling was standardised by selecting enamel sections from the same region in the tooth, i.e. the central part of the tooth crown. The investigated fragment should therefore have mineralised between the age of two and eight, and represents a weighted mean of the food and drink ingested over multiple years. Additional sampling and methods details can be found in Montgomery *et al*.^43^.

Tooth enamel bioapatite was precipitated as silver phosphate (Ag_3_PO_4_) following a customised version of the procedures described in Dettman *et al*.^49^. Its isotopic composition was measured via Thermal Conversion Mass Spectrometry following the principles outlined in Gehre and Strauch^50^. Enamel chips were crushed to a homogenous fine powder in an agate mortar and pestle, and about 8–10 mg of this powder was dissolved in 2ml HF for 24h. Following the results in Grimes and Pellegrini^51^, no pre-treatment protocol was performed to clean the samples of potential organic contaminants. The solution containing dissolved phosphate ions was separated from the precipitated CaF_2_ by centrifugation, and the solid CaF_2_ rinsed three times with MilliQ water, the rinsing water being also added to the phosphate solution. The liquid was subsequently mixed with 2M NH_4_OH and AgNO_3_ to precipitate Ag_3_PO_4_, which crystals were separated by centrifugation from the solution and dried out overnight at 60 °C. An aliquot of 250–350 μg of these homogenised crystals was loaded into silver capsules and combusted in a Thermo Finnigan^TM^ High Temperature Conversion Elemental Analyser, coupled with a stream of helium to a Thermo Finnigan^TM^ Delta Plus XL mass spectrometer set at a temperature of 1450 °C.

The results given in the SI for each sample are the average of two to five repeated measurements. The average reproducibility of the isotopic measurements for each sample was 0.13‰ ± 0.09 (1σ). Raw data were corrected with repeatedly measured standard materials, regularly analysed within the run in the same conditions as the samples, to compensate for drifts in both the mass spectrometer and the peripheral. Shifts from the assigned values of the standards were applied to the value of the measured sample^52^. The correction applied to phosphate oxygen isotope measurements is not free of complications, given the lack of internationally recognised standards^53^,^54^ and the poor uniformity of the precipitation methods and calibration approaches employed in the different laboratories. For this project, raw data were corrected against widely-used silver phosphate materials (TU1 = 21.1‰ and TU2 = 5.5‰) that ‘bracketed’ the composition of all the samples investigated in this study (i.e. they have lower and higher values). These working standards were reported in Vennemann *et al*.^55^. A two-point linear correction based on these standards was applied, as also recommended by Meier-Augenstein^53^. A third reference material, Ag_3_PO_4_ precipitated in large amounts in our lab from the phosphorite rock international standard SRM 120c (formally NBS 120c), was instead used as an external check for the calibration. Although frequently used as a reference standard for phosphate oxygen isotopes because it is a suitable inorganic analogue to bioapatite, this material is technically only a reference standard for its chemical content and not for its isotopic composition. Additionally, it has to be precipitated (i.e. manipulated) as Ag_3_PO_4_ in each laboratory before isotopic analysis. It also has a quite large interval of reported values ranging from 19.9‰ to 22.6‰, with a mean of 21.7‰ ± 0.5‰^20^. Finally, its rather ^18^O-enriched value, outside the range of most phosphate values for humans from northern Europe and Britain, made it unsuitable to calibrate our dataset. Within this study, our SRM 120c Ag_3_PO_4_ working standard gave a range of 22.1 ± 0.3‰ (n = 72), in line with reported values.

SRM 120c was also employed in this study to check the accuracy and reproducibility of the precipitation protocol by including samples of this standard in each precipitation batch together with the unknown samples. The variation observed in the composition of this material precipitated with the samples was approximately 0.2‰ (n = 8). Two additional materials, an inorganic apatite (HAP), and an in-house matrix-matching bioapatite enamel standard (BES) were also repeatedly used within the batches. The values obtained for these materials are 17.1 ± 0.3‰ (n = 6) and 17.0 ± 0.2‰ (n = 9), respectively, suggesting an overall good reproducibility of the precipitation method employed in this study.

As mentioned, because of the lack of an internationally recognised standard for phosphate oxygen isotope methods and commonly agreed protocols for precipitation and calibration, other laboratories might use different ways of correcting their *δ*^18^O_p_ values. This makes comparisons of different datasets problematical. By using different calibration methods (1-point *vs* multiple point calibrations), for example, we can predict that the average possible shift between data calibrated in different ways can be between 0.3‰ and 0.5‰, but can reach 1‰, with the highest impact on the extreme values (*i.e*. those that have the most enriched or the most depleted *δ*^18^O compositions). For this reason, only data from the Beaker People Project were used to create the geostatistical model presented in this paper.

## Additional Information

**How to cite this article**: Pellegrini, M. *et al*. Tooth enamel oxygen “isoscapes” show a high degree of human mobility in prehistoric Britain. *Sci. Rep*. **6**, 34986; doi: 10.1038/srep34986 (2016).

## Supplementary Material

Supplementary Information

Supplementary Table S1
