# Supplementary material for: Tooth enamel oxygen “isoscapes” show a high degree of human mobility in prehistoric Britain
Source: Sci Rep. 2016 Oct 7;6:34986. doi: 10.1038/srep34986 (PMC5054518; doi:10.1038/srep34986)
Supplement: Supplementary Information [file srep34986-s1.pdf]

Tooth enamel oxygen “isoscapes” show a high degree of human mobility in prehistoric Britain

Maura Pellegrini, John Pouncett, Mandy Jay, Michael Parker Pearson, Michael P. Richards

Supplementary Information

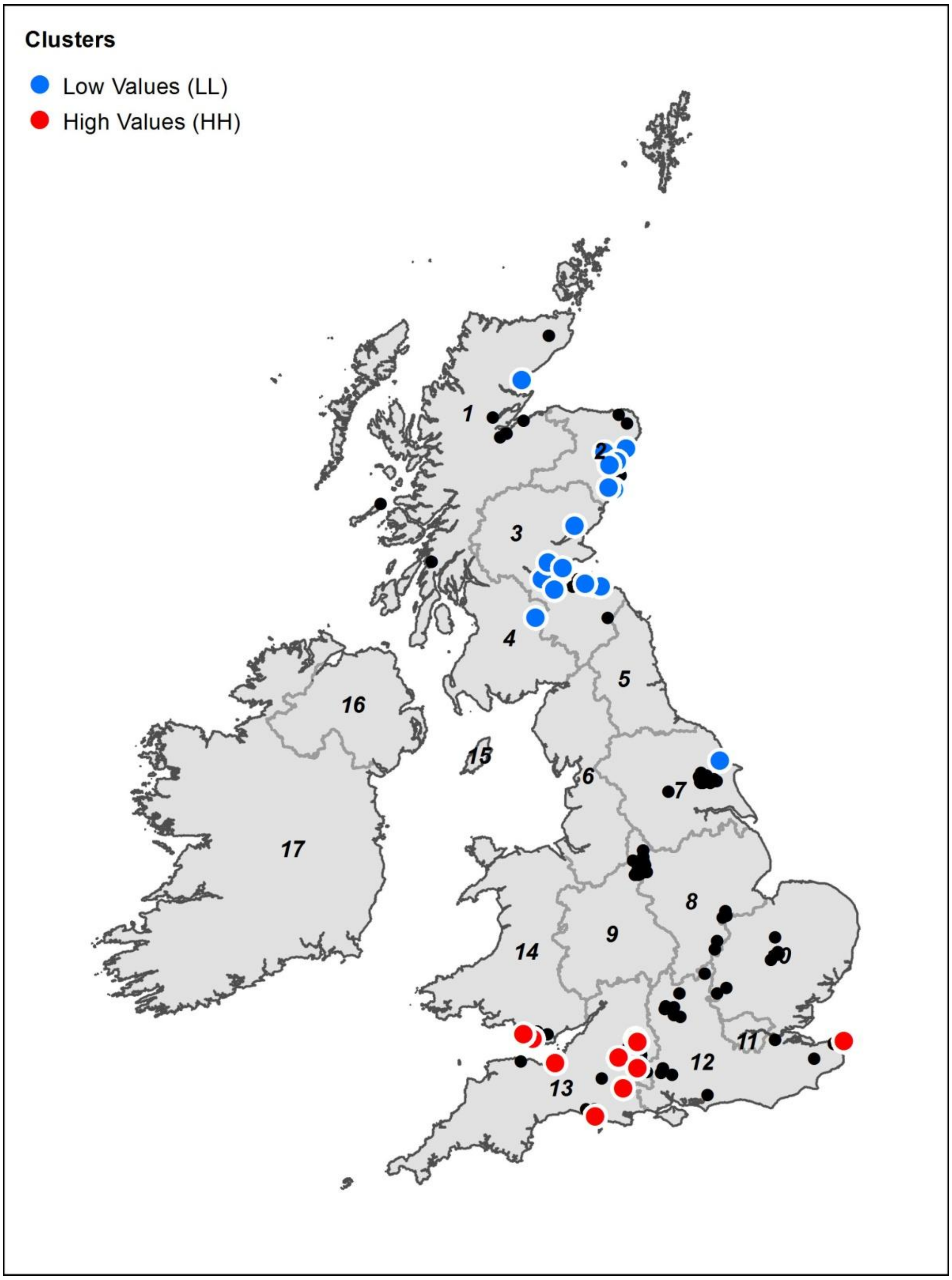

Fig. S1: Statistically significant clusters of high and low $\delta^{18}O_p$ values at 95% confidence level, identified on the basis of the calculation of the Anselin's Local Moran's I test statistic (Region codes as in Fig.3). Cluster/Outlier Analysis carried out using ArcGIS for Desktop 10.3.1 (http://desktop.arcgis.com/en/arcmap/10.3/tools/spatial-statistics-toolbox/cluster-and-outlier-analysis-anselin-local-moran-s.htm) © EuroGeographics for the administrative boundaries
